# Medical Management of Isolated Partial Anomalous Pulmonary Venous Connection With Symptomatic Right Ventricular Failure and Pulmonary Hypertension

**DOI:** 10.7759/cureus.62779

**Published:** 2024-06-20

**Authors:** Myoung Hyun Choi, Frances Greathouse, Amir Darki

**Affiliations:** 1 Internal Medicine-Pediatrics, Loyola University Medical Center, Maywood, USA; 2 Internal Medicine, Loyola University Medical Center, Maywood, USA; 3 Cardiology, Loyola University Medical Center, Maywood, USA

**Keywords:** pulmonary hypertension, treatment of pulmonary arterial hypertension, heart failure with preserved ejection fraction, partial anomalous pulmonary venous connection, adult congenital heart disease (achd)

## Abstract

Partial anomalous pulmonary venous connection (PAPVC) is a rare congenital heart disease in which one or more pulmonary veins drain into the systemic venous circulation. The abnormal connection between the pulmonary vein and the right atrium can result in a right-sided volume overload due to a left-to-right shunt, followed by eventual right-sided pressure overload and right ventricular failure. PAPVC is usually associated with an atrial septal defect but can present as an isolated finding. We present a case of isolated PAPVC resulting in right heart failure and predominantly pre-capillary pulmonary hypertension. We discuss the challenges in the diagnosis and medical management of isolated PAPVC and highlight the clinical and hemodynamic indications for pulmonary vasodilators and diuretics.

## Introduction

Anomalous pulmonary venous connection (APVC) is a rare congenital defect accounting for less than 1% of all congenital heart diseases in which one or more pulmonary veins drain into the systemic venous circulation. It can be classified into total APVC (TAPVC) where all four pulmonary veins fail to terminate in the left atrium (LA) or partial APVC (PAPVC) where only one or more pulmonary veins return to the right heart. TAPVC presents with cyanosis shortly after birth and requires urgent surgical intervention. On the other hand, PAPVC can present later in life with heart failure symptoms, elevated right ventricular filling pressures, and pulmonary hypertension (PH) due to left-to-right shunting [1]. The age and severity of symptoms at presentation correlate with the degree of shunt, the number of anomalous venous return, and the presence of other cardiac anomalies [2]. For example, a single anomalous pulmonary venous connection is unlikely to result in significant volume overload. PAPVC is often associated with an atrial septal defect (ASD) but can be isolated [3]. Arrhythmias are also commonly seen in PAPVC. Atrial fibrillation and atrial arrhythmias were confirmed in 21.9% of patients with PAPVC in one retrospective study [2] explained by ectopic foci arising from non-pulmonary vein areas like the superior vena cava (SVC) [4,5].

In the diagnosis of PAPVC, transthoracic echocardiography (TTE) is not always able to identify abnormal venous connections but can visualize indirect signs of PAPVC including dilated right ventricle (RV) and elevated RV pressure. Cross-sectional imaging with cardiovascular magnetic resonance (CMR) or cardiac computed tomography (CCT) is the preferred imaging modality to identify anomalous venous connections. A transesophageal echocardiogram (TEE) can be used to rule out interatrial septal defects. Surgical correction is recommended for patients with impaired functional capacity, RV enlargement, large net left-to-right shunt with Qp:Qs ≥1.5:1, pulmonary artery (PA) systolic pressure less than 50% of the systemic pressure, and pulmonary vascular resistance (PVR) less than one-third of the systemic vascular resistance (SVR) [2,3]. For cases with concomitant ASD with irreversible shunt reversal and Eisenmenger physiology, lung transplantation or heart-lung transplantation is considered [6,7]. Medically, clinical observation and/or pulmonary artery vasodilator therapy with guanylate cyclase inhibitors, endothelin receptor antagonists, and phosphodiesterase 5 inhibitors have been reported [7,8]. However, little is reported on the medical management of isolated PAPVC outside of pulmonary vasodilators. Here, we report a case of isolated PAPVC and discuss the therapeutic options presented to us as well as their respective indications.

## Case presentation

A 68-year-old female of Hispanic origin, recently immigrated to the United States, with a past medical history of surgically corrected ASD, heart failure with preserved ejection fraction, PH, atrial fibrillation, and chronic kidney disease presented to the emergency department at an outside hospital with worsening dyspnea at rest and on exertion. Before this, she had been experiencing flu-like symptoms and a dry cough for a week. She was adherent to her home medications, which included amiodarone, digoxin, empagliflozin, furosemide, sacubitril-valsartan, spironolactone, and tadalafil. She had never used tobacco products and did not consume alcohol. She had not been limiting her water intake. 

**Figure 1:**
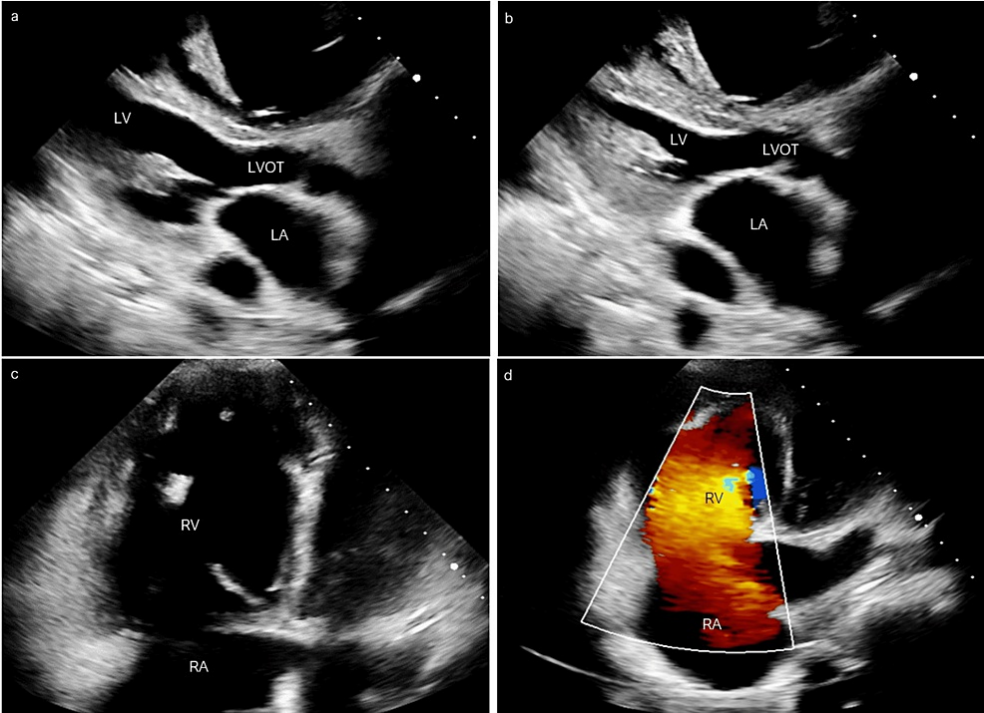
Transthoracic echocardiogram, parasternal long-axis view. (a, b) Show severely underfilled LV at diastole with normal size and EF, respectively. (c, d) Show severely dilated RV with mildly reduced function and high flow through the right side of the heart, respectively. LV, left ventricle; EF, ejection fraction; RV, right ventricle; LA, left atrium; RA, right atrium; LVOT, left ventricular outflow tract.

In the emergency department, her blood pressure was 113/52 mmHg, heart rate 77 beats per minute, and oxygen saturation 95% on room air. Physical examination revealed irregularly irregular rhythm, 3+ bilateral pitting edema, bibasilar inspiratory crackles, and ascites. Chest x-ray showed diffuse patchy opacities in both lungs. Her labs were remarkable for B-type natriuretic peptide (BNP) greater than 1,000 pg/mL. She was initiated on intravenous furosemide with improvement in her symptoms. TTE showed a small and underfilled left ventricle (LV) with normal size and ejection fraction (EF) (Figures 1a, 1b). The left atrial volume was 42.4 mL and the left atrial volume indexed for body surface area was 24.7 mL/m^2^. The RV was severely dilated with mildly reduced function and apparent high flow through the right side of the heart concerning an interatrial shunt or anomalous pulmonary vein drainage (Figure 1c). The PA systolic pressure was estimated to be 70 mmHg and the right atrium (RA) pressure was 15 mmHg with no interatrial shunt on the bubble study (Figure 1d). The patient was transferred to a tertiary center for further management and work-up of possible anomalous pulmonary venous return and other intracardiac shunt.

After significant diuresis, the patient underwent a right heart catheterization which revealed a severely elevated RA pressure of 18 mmHg, PA pressure of 88/23 mmHg with a mean of 45 mmHg, elevated PVR of 2.47 woods units (WU), mean pulmonary capillary wedge pressure (PCWP) of 15 mmHg equivalent to the LV end-diastolic pressure, cardiac output (CO) of 10.95 L/min, and cardiac index (CI) of 6.3 L/min/m^2^, suggesting predominantly pre-capillary PH (PCWP of 15 mmHg or less, PVR >2 WU, and mPAP >20 mmHg) [9]. The ratio of pulmonary (Qp) and systemic (Qs) flow (Qp:Qs) was 1.73. A vasoreactivity study with inhaled nitric oxide reduced the PVR to 1.06 WU but increased the Qp:Qs ratio to 2.95 with a significant left-to-right shunt of 11.31 L/min and a nearly absent right-to-left shunt of 0.1 L/min. The CO and CI were also reduced to 5.76 L/min and 3.32 L/min/m^2^, respectively. Given predominantly pre-capillary PH, a fluid challenge was not performed [10].

**Figure 2:**
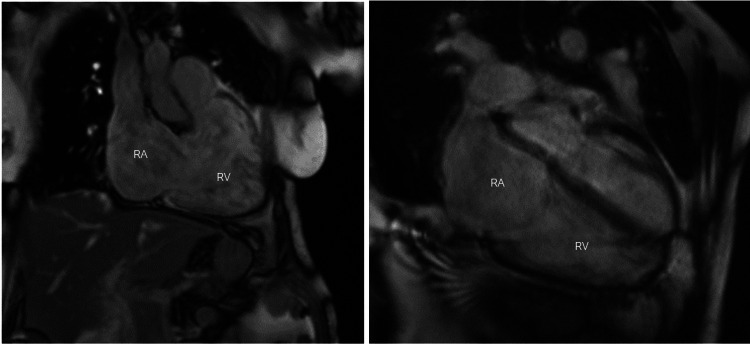
Cardiac magnetic resonance imaging Cardiac MRI showing dilated RA and RV. The RV is severely dilated in size with normal systolic function (RVEF = 54%). The anomalous left pulmonary venous drainage into the RA could not be visualized in this study. MRI, magnetic resonance imaging; RA, right atrium; RV, right ventricle; EF, ejection fraction.

CMR imaging (Figure 2) revealed a prominently dilated coronary sinus, a severely dilated RV and a dilated RA, and anterior and inferior RV insertion site fibrosis suggestive of anomalous pulmonary vein drainage though not visualized in the study. There was no clear evidence of an ASD.

**Figure 3:**
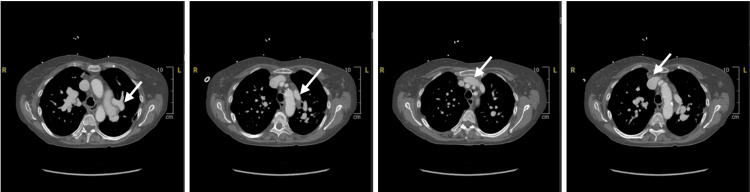
Cardiac CT of left superior pulmonary vein Cardiac CT demonstrating left superior pulmonary venous drainage into the left brachiocephalic and ultimately into the SVC (left to right). CT, computed tomography; SVC, superior vena cava.

**Figure 4:**
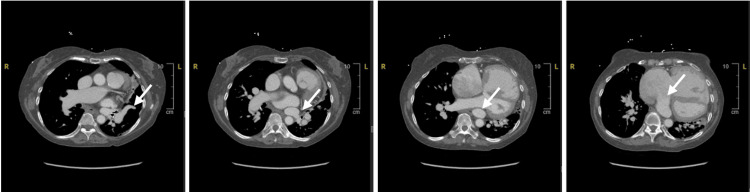
Cardiac CT of the left inferior pulmonary vein Cardiac CT demonstrating left inferior pulmonary venous drainage into the coronary sinus and ultimately into the RA (left to right). CT, computed tomography; RA, right atrium.

Further imaging with CCT revealed anomalous pulmonary venous return with two right pulmonary veins draining into the LA, the left superior pulmonary vein traveling superiorly into the dilated right SVC (Figure 3), and the left inferior pulmonary vein draining into the coronary sinus which was dilated and draining into the RA (Figure 4). The pulmonary artery was dilated at 4.1 cm.

Though her invasive hemodynamics were consistent with predominantly pre-capillary PH, tadalafil was discontinued due to increased left-to-right shunt and reduced CO with inhaled nitric oxide. Medical management with intravenous diuresis was optimized. Her sacubitril-valsartan, digoxin, and empagliflozin were discontinued. She was started on apixaban for stroke prevention. No acute surgical intervention was indicated, and the decision to continue medical therapy to maintain euvolemia and rate control while undergoing further surgical workup was made. Once reaching clinical euvolemia, she was discharged on oral diuretics with scheduled follow-up with Cardiothoracic Surgery and Cardiology.

## Discussion

Isolated PAPVCs are difficult to diagnose as they are often clinically silent and become symptomatic in late adulthood with the development of a significant left-to-right shunt (Qp:Qs ≥ 1.5). They are typically identified incidentally on TTE while undergoing other cardiopulmonary workup. While TTE can identify anomalous pulmonary veins, it is often limited and requires subsequent cross-sectional imaging with CMR or CCT to better delineate pulmonary venous connection. TEE is often obtained to rule out interatrial septal defects [6].

Medical management of heart failure and PH in patients with isolated PAPVC remains a significant challenge. Surgical repair is the mainstay of corrective treatment for PAPVC when there is functional impairment, RV enlargement, large net left-to-right shunt (e.g., Qp:Qs ≥ 1.5), PA systolic pressure less than 50% of systemic pressure, and PVR that is less than one-third of SVR [6]. However, most patients will benefit from medical therapy to maintain euvolemia and rate and/or rhythm control for concurrent arrhythmia as a bridge to surgery or as a destination therapy when surgery is not an option.

Medical therapy for PH in a patient with PAPVC should be individualized based on several factors including clinical symptoms, the number of anomalous venous connections, hemodynamics, degree of RV failure, degree of left-to-right shunt, presence of shunt reversal, and presence of an ASD. Pulmonary vasodilators are typically used in patients with Eisenmenger syndrome when the development of shunt reversal (Qp:Qs < 1) precludes surgical repair due to high surgical risk. Pulmonary vasodilators are also used in patients with either isolated pre-capillary PH (PCWP ≤ 15) or post-capillary PH (PCWP > 15) with a pre-capillary component (PVR > 2) if there is evidence of subsequent improvement in hemodynamic parameters [7-9,11].

In our patient, a decrease in CO and an increase in left-to-right shunt with inhaled nitric oxide precluded the use of pulmonary vasodilators. Instead, her oral diuretic regimen consisting of a mineralocorticoid receptor antagonist and loop diuretic was optimized, leading to symptomatic improvement. On her outpatient follow-up, she continued to experience significant improvement in her symptoms, and a decision was made to monitor clinically while continuing her outpatient surgical workup.

## Conclusions

Isolated PAPVC poses a significant diagnostic challenge as it is usually clinically silent and manifests later in life. At the time of diagnosis, patients often have RV dysfunction that is significant enough to cause permanent cardiopulmonary remodeling with symptoms of congestive heart failure and PH. Diagnostic tools such as TTE, CMR, and/or CCT are essential. While surgical repair is the mainstay of treatment, tailored medical therapy can serve as a bridge to surgical repair or as destination therapy. Pulmonary vasodilators are used in patients with shunt reversal or pre-capillary PH. However, they may be contraindicated in patients whose vasoreactivity test shows worsening left-to-right shunt and/or reduction in CO. In isolated PAPVC with predominantly pre-capillary PH and RV failure, oral diuretics may be a more appropriate option to decrease RV filling volume and PCWP, thereby preventing further pulmonary venous congestion and RV failure.
